# Retroperitoneal fibromatosis presenting as a mesenteric mass: A case report

**DOI:** 10.1097/MD.0000000000018799

**Published:** 2020-04-24

**Authors:** Jianchun Xiao, Wenzhe Zhou

**Affiliations:** b Department of Hematology, Chinese Academy of Medical Sciences and Peking Union Medical College Hospital, Beijing; a Department of General Surgery, Peking Union Medical College Hospital, China.

**Keywords:** case report, intra-abdominal fibromatosis, mesenteric occupancy, small intestine

## Abstract

**Rationale:**

Fibromatoses or desmoid tumors are relatively rare tumors derived from the musculoaponeurotic system. This tumor has no specific clinical symptoms and it is sometimes misdiagnosed as other diseases such as gastrointestinal stromal tumors (GISTs).

**Patient concerns:**

A 28-year-old man visited Peking Union Medical College for a tangible abdominal mass without abdominal pain or distention.

**Diagnoses:**

Considering the imaging characteristics and clinical manifestation, this mass was primarily diagnosed as GIST before surgery. During the surgery, the occupancy was found under the ileocecal mesentery, with grayish white appearance, tough texture, and poor mobility, which was not consistent with the character of the GIST. After the surgery, pathological examination and individual immunohistochemistry results demonstrated that the lesion was compatible with the diagnosis of retroperitoneal fibromatosis with purulent inflammation of chronic lymphadenitis.

**Interventions:**

Therefore, we decided to perform tumor mass resection, right colon resection, partial duodenum resection, and intestinal anastomosis on laparotomy, but the right ureter was retained. After excision of the tumor, the ends of the intestine segment were continuously sutured.

**Outcomes:**

The patient experienced no intraoperative or postoperative complications, and was discharged 3 days after surgery. Periodic follow-up physical examinations such as the abdominal ultrasound and computed tomography were performed each 3 months, and no evidence of recurrence was observed during the whole 12 months.

**Lessons:**

To sum up, intra-abdominal fibromatosis is an extremely rare tumor that must be differentiated from other tumors of the digestive tract, and pathological and immunohistochemical examination is a critical part of the diagnosis. Early diagnosis of fibromatosis is essential for the outcome. Extensive resection of the mass minimizes the risk of relapse.

## 1 Introduction

Intra-abdominal fibromatosis is a rare tumor that accounts for approximately 0.03% of all neoplasms. This tumor is relatively benign as it may invade local tissue but hardly metastasize distantly.^[1–3]^ The etiology of fibromatosis is unclear and it is usually connected with endocrine factors or trauma.^[2]^ Because these tumors are usually asymptomatic, there are no clear guidelines for treatment. The intestinal mesentery is usually the origin of intra-abdominal fibromas, but rare fibromas locate in abdominal ligands and the colon.^[3]^ Surgical resection with a wide margin is the preferred selection of treatment in most cases. For tumors that may relapse or are unable to be resected, medical therapy is recommended.^[4]^

We herein report a case of duodenum-derived fibromatosis presenting as an abdominal mass, which was preoperatively considered to be a gastrointestinal stromal tumor with no particular symptom or trauma history.

## 2 Case report

A 28-year-old young male suffered from a tangible abdominal mass in the right middle abdomen and was referred to Peking Union Medical College Hospital. Physical examination revealed a palpable mass on the right side of umbilical cord with no tenderness.

**Figure 1:**
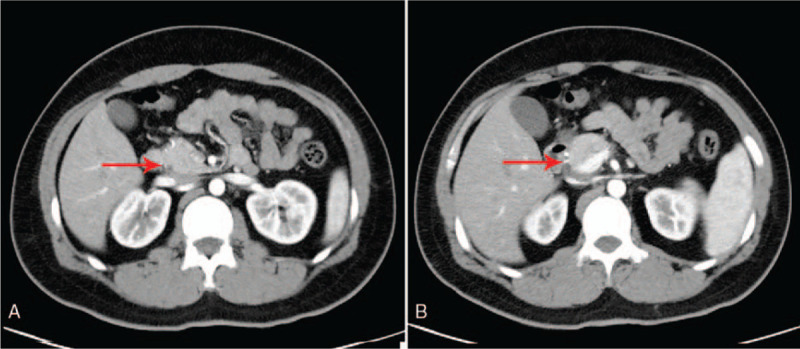
. Abdominal enhancement computed tomography scan: Red arrows indicate a homogenous mass measuring 7.1 cm × 7.6 cm with uniform enhancement.

Abdominal enhancement computed tomography (CT) revealed (Fig. 1A and B) a mass with uniform soft tissue density and inhomogeneous enhancement in lower abdomen; the maximum cross-sectional area of the mass was about 7.1 cm × 7.6 cm. The mass was considered to be small intestinal stromal tumor while fibromatosis could not be excluded. As the imaging findings were atypical, further exploration with surgery was necessary.

**Figure 2:**
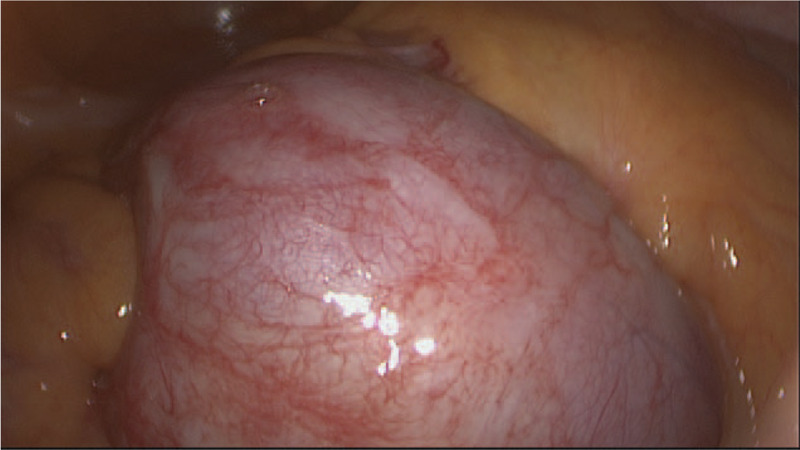
. Laparoscopic image presenting the resection of the mass: The bases of the tumor infiltrated the intestine and ileocecal mesentery region and closely connected to the ureter in the retroperitoneum. Tumor mass resection, right colon resection, partial duodenum resection, and intestinal anastomosis were performed.

On laparotomy, 7.1 cm × 7.6 cm sized round tumor located under the ileocecal mesentery, with grayish white appearance, tough texture, and poor mobility was found. The base of the tumor was closely connected to the right ureter in the retroperitoneum and was adhered to the small intestine and ileocecal mesenteric region in the abdominal cavity, which was extremely difficult for separation and resection (Fig. 2). Then tumor mass resection, right colon resection, partial duodenum resection, and intestinal anastomosis were performed while the right ureter was retained. After excision of the tumor, the ends of the gastric segment were continuously sutured. No intraoperative complications occurred. The preoperative and intraoperative conditions were stable, and 3 days after the surgery, the patient was discharged.

**Figure 3:**
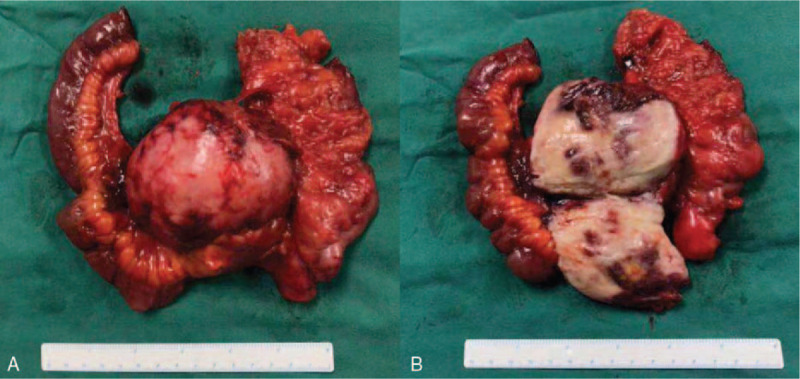
. Gross pathology of the fibromatosis. (A) The fibromatosis invaded ileocecal intestine and mesenterium. (B) Transaction of the fibromatosis. The mass substance measuring 7.1 cm × 7.6 cm had a regular shape and rubbery, hard consistency.

**Figure 4:**
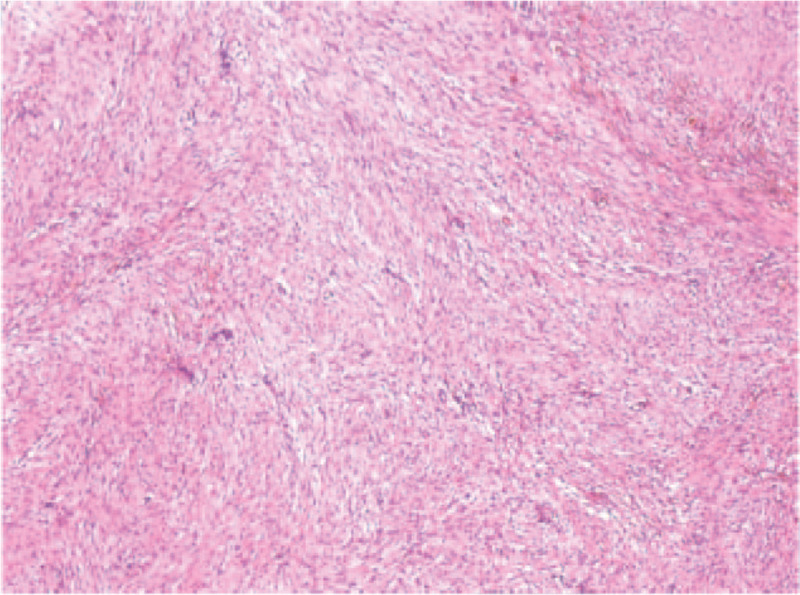
. Histological examination (hematoxylin and eosin staining) of the fibromatosis. Photomicrograph of the histologic specimen revealing an abnormal proliferation of fibroblasts, and individual immunohistochemical examination demonstrating β-catenin (+), vimentin (+), smooth muscle actin (−), S100 (scattered +), Ki67 (index 5%), DOG1 (−), CD34 (−), CD117 (−), and desmin (−).

Gross pathology revealed that the tumor invaded the whole small intestinal and ascending colonic wall (Fig. 3A and B). Pathological examination revealed transitional hyperplasia of epithelial cells and spindle cells atypia in the muscular layer of intestinal wall whereas the mucosa layer remained intact (Fig. 4). Individual immunohistochemistry results demonstrated that the tumor was positive for β-catenin, smooth muscle actin (SMA), and S100 (scattered+), but negative for CD34, CD117, DOG-1, Ki-67, and desmin, which were compatible with the diagnosis of retroperitoneal fibromatosis with purulent inflammation of chronic lymphadenitis.

The patient underwent periodic follow-up physical examinations, in which the abdominal ultrasound and CT were performed each 3 months, and no evidence of recurrence was observed during the whole 12 months.

## 3 Discussion

Fibromatoses or desmoid tumors are relatively rare tumors derived from the musculoaponeurotic structures.^[5]^ Although there is no specific etiology, some risk factors are known to be associated with this tumor, including trauma, surgery, pregnancy, use of contraceptives, genetic mutation, family history of desmoid tumor, familial adenomatous polyposis, and Gardner syndrome.^[6]^ The involved scope of fibromatosis ranges from the gastrointestinal tract to the liver, and retroperitoneum.^[3]^ As for pathological features, spindle cells are common in desmoid tumor. For immunohistochemical features, vimentin and β-catenin are positive, whereas SMA (including S100, CD117, and CD34) is immunonegative.^[6]^ But retroperitoneum-derived fibromatosis with immunopositive SMA and S100 that almost infiltrated the whole intestinal wall is rather rare.

The main clinical manifestation of desmoid tumors, such as vomiting, abdominal distension, difficulty in defecation due to intestinal obstruction, and hydronephrosis coursed by ureteral involvement,^[3]^ were determined by its size and surrounding anatomical structures.^[7]^ But in our case, although the large-sized mass involved the ureter and bowel, there were no intestinal obstruction or hydronephrosis in this patient, perhaps related to its retroperitoneum origin rather than from ureter and intestine.

As above, in about 30% of patients, fibromatoses are asymptomatic until it could be found in a regular clinical examination.^[8]^ Retroperitoneal fibrosis frequently presents as a mass involving small bowel mesentery, so it is extremely difficult to distinguish it from inflammatory lesions, mesenteritis, and several benign gastrointestinal tumors.^[9]^ The presurgical diagnosis of intra-abdominal fibromatosis depends mainly on imaging. On ultrasound, fibromatosis showed variable echogenicity with a smooth, clear border.^[10]^ On CT, this tumor usually presents as a slightly lower density with uneven enhancement.^[11]^ Therefore, the appearance of intra-abdominal fibromatosis on a CT scan is untypical. However, pathology is still the gold standard to provide a concrete diagnosis of intra-abdominal fibromatosis. Under microscopy, spindle cells arranged in bundles, surrounded by large amounts of collagen, could be seen. Immunohistological examination always indicates the positive expression of vimentin and SMA.^[12]^

Treatments include surgical resection, radiation therapy, or a combination of both. Although in this case, the tumor has local invasive growth, its outer membrane makes it possible to determine the effect of intraoperative scar or the surrounding tissue and achieved R0 resection. However, it still has a strong tendency to local recurrence, so routine postoperative adjuvant radiotherapy is necessary.^[13]^ For relapsed and unresectable tumor, anti-hormone therapy, nonsteroidal anti-inflammatory drugs, and other drugs can be used.^[14]^ The patient presented in this study has a high risk of recurrence because the tumor was much larger than 50 mm in diameter, so the strict follow-up of this patient should be ensured in the next 5 years.^[15]^

## 4 Conclusion

It is usually incidental and challenging to diagnose fibromatosis especially in the case with no clinical symptoms. The retroperitoneum-originated fibromatosis invaded the ileocecal mesothelium and ureter was positive for S100 protein, which is very rare. This study provided some suggestions for diagnosis and treatment of anonymous abdominal mass, exploratory laparotomy is necessary in several cases, and pathological and immunohistochemical examination is the key to diagnosis. Early diagnosis of fibromatosis is essential for the outcome. Extensive resection of the mass can minimize the risk of relapse according to the existing literature.

Postoperative pathological examination confirmed that it was a fibromatosis. The operative decision was correct and the effect was satisfactory. No complications occurred during postoperative follow-up.

## Acknowledgments

The authors would like to appreciate the patient who consented to disclose their medical records and answered our review calls.

## Author contributions

QQ conceived the idea of the study; XJ collected the information; ZW and XJ interpreted the results and wrote the paper.

**Conceptualization:** Qiang Qu.

**Data curation:** Wenzhe Zhou.

**Resources:** Jianchun Xiao.

**Writing – original draft:** Wenzhe Zhou.

**Writing – review & editing:** Wenzhe Zhou and Jianchun Xiao.
